# Supplementary material for: Selective activation of FZD7 promotes mesendodermal differentiation of human pluripotent stem cells
Source: eLife. 2020 Dec 17;9:e63060. doi: 10.7554/eLife.63060 (PMC7759383; doi:10.7554/eLife.63060)
Supplement: Supplementary file 4. [file elife-63060-supp4.docx]

Supplementary File 4: Plasmids available upon request

| **Plasmid ID** | **Description** | **Mammalian cell drug selection** | **Bacterial cell drug selection** |
| --- | --- | --- | --- |
| pKW483 | Mammalian expression vector encoding F7L6-sc under control of CMV promoter. | Puromycin | Ampicillin |
| pKW522 | Mammalian expression vector encoding F7L6 under control of CMV promoter. The complete amino acid sequence of F7L6 is provided in Figure 1—figure supplement 1. | Puromycin | Ampicillin |
